# Medication adherence to antiretroviral therapy among newly treated people living with HIV

**DOI:** 10.1186/s12889-018-5731-z

**Published:** 2018-07-04

**Authors:** Yang Yu, Dan Luo, Xi Chen, Zhulin Huang, Min Wang, Shuiyuan Xiao

**Affiliations:** 1 Department of Social Medicine and Health Management, Xiangya School of Public Health, Central South University, Changsha, 410078 China; 2 Hunan Provincial Center for Disease Prevention and Control, Changsha, China; 3 Changsha Center for Disease Prevention and Control, Changsha, China; 4 HIV/AIDS Research Institute, The First Hospital of Changsha, Changsha, China

**Keywords:** People living with HIV, Antiretroviral therapy, Medication adherence

## Abstract

**Background:**

Free antiretroviral therapy has been implemented in China since 2004, but adherence to antiretroviral therapy among people living with HIV is suboptimal. The effectiveness of antiretroviral therapy is subject to medication adherence, which decreases with prolonged treatment times. The aim of this study was to investigate medication adherence and related factors among people living with HIV with newly initiated antiretroviral therapy.

**Methods:**

This observational study was conducted in consecutive samples of people living with HIV who had newly initiated antiretroviral therapy. Participants were recruited between March 1, 2013, and August 31, 2014, from the local Center for Disease Control and Prevention and Infectious Disease hospital in a capital city in central China. A standard set of questionnaires was adopted, including the Community Programs for Clinical Research on AIDS Antiretroviral Medications and Self-Report Questionnaire (CPCRA), the Patient Health Questionnaire-9 (PHQ-9) and the 7-item Generalized Anxiety Disorder Scale (GAD-7). T-test, Chi square test and multivariate logistic regression analysis with backward stepwise were performed to explore factors that might influence medication adherence.

**Results:**

Of the 207 participants, 85.5% of the participants (177/207) were categorized with good adherence, and 14.5% (30/207) with poor adherence. The multivariate logistic regression analyses showed that participants with positive depression (OR = 5.95, 95% CI: 2.34–15.11) and without disclosure of their HIV status to others (OR = 2.62, 95% CI: 1.06–6.50) were more susceptible to poor adherence.

**Conclusions:**

One-sixth of the participants reported suboptimal medication adherence within the first 6 months. Factors associated with poor adherence included non-disclosure of their HIV status, had positive depression. Tailored interventions, such as effective psychological coping strategies, should be implemented for people living with HIV with newly initiated antiretroviral therapy to improve their medication adherence.

## Background

Antiretroviral therapy (ART) has proven to be the most effective treatment method for people living with HIV [1, 2], its effectiveness critically depends on the extent of individual medication adherence [3–5]. When the rate of adherence to medication is as high as 95%, the viral suppression rate approaches 78%. However, when the rate of adherence is reduced to 80%, there is a dramatic reduction in the viral suppression rate, which can be as low as 20%. The adherence rate of medication should be maintained at 95% or above to optimize antiviral outcomes and enhance viral suppression [6, 7]. Additionally, failure to maintain the blood concentrations of the medications is a common and serious problem, data indicated that dosing time adherence was significantly associated with lower viral load, and that was also associated to elevated CD4 cell count [8, 9]. Poor adherence to medication has been confirmed to be an important risk factor for the emergence of drug-resistant HIV strains, which can be transmit to others [10, 11]. Poor medication adherence not only endangers individual health but also increases of transmission with detectable viral loads, brings difficulties in treatment, leading to severe public health problems.

The “Four Frees and One Care” policy was promulgated in China in 2004. As a result, more than 420,000 AIDS patients received antiretroviral therapy by June 2016 [12]. HIV/AIDS is becoming a manageable chronic condition, and adherence to ART, especially adherence to medication, is the focus of many researchers. Medication adherence is defined as taking all medications at the appropriate time with the appropriate dosage as prescribed by the physician [13, 14]. Although there is no standard method of measurement, self-report questionnaires are widely used due to their simplicity and validity. Their results are consistent with objective measurement methods, such as electronic drug concentration monitoring and plasma drug concentration monitoring [15]. Zhou et al. conducted a meta-analysis of medication adherence among patients receiving antiviral therapy in China from 1985 to 2015 [16] and found that the 90% medication adherence rate was 77.61%, which was lower than the rates reported by similar studies conducted in other countries [17–19]. This finding suggested a greater need to pay attention to this group of people in China.

Adherence to medication is a complicated behavior that is affected by disease-related, cognitive and psychological, and provider-related factors [17, 20]. Disease-related factors include symptoms [19], the disease course [21, 22], and side effects [22]. Cognitive and psychological factors include self-efficacy [23], the depression or anxiety status [24], and knowledge of medication [17]. Provider-related factors include patient-provider relationships [25], the provision of limited instructions [21, 26], and low-quality services [26].

Previous studies had several limitations in assessing adherence to medication. First, the assessment was based solely on the self-reported quantity of drugs taken by the patients but not on the timing of drug intake. In addition, neglect of the time difference of drug intake may lead to an overestimation of adherence [15]. Second, the stage of ART was not specified. As the treatment duration progresses, the probability of non-adherence increases [27]. Therefore, high priority must be given to the assessment of adherence behavior as well as any necessary intervention for non-adherence during the early stages of treatment.

Optimizing adherence, especially in the early stages of treatment, is crucial to ensure long-term immunological and virological success [27]. For people living with HIV who have newly initiated antiretroviral therapy, accurately evaluating their medication adherence and identifying the factors associated with adherence can help providers to predict their prognosis and adopt timely interventions to facilitate and maintain the optimum effects of antiviral therapy. Given the above considerations, this study aimed to investigate medication adherence with respect to both quantity and timing and the factors related to adherence among people living with HIV with newly initiated antiretroviral therapy and to provide evidence for formulating individualized interventions to improve treatment outcomes.

## Methods

### Study setting

This study was conducted at the Center for Disease Control and Prevention (CDC) and the Infectious Disease Hospital of a capital city in central China. These two institutions were designated by the government to provide HIV/AIDS care, including free HIV testing, diagnosis, management, and dispensation of free medication for registered people living with HIV.

### Study participants

This study was based on an observational research project that evaluated changes in emotional problems and disease course among newly diagnosed people living with HIV. HIV-seropositive individuals who were newly diagnosed and registered in the Chinese HIV/AIDS Comprehensive Response Information Management System between March 1, 2013, and August 31, 2014, were recruited. Participants were sequentially enrolled if they 1) were 18 years or older, 2) had lived in the sampled city for more than 6 months, 3) were without cognitive impairment, and 4) were willing to participate in the face-to-face interview. All eligible participants were followed-up for 1 year, and the face-to-face interview was conducted within 3 months after the antiretroviral therapy regime was established.

During the sample enrollment interval (from March 1, 2013, to August 31, 2014), 855 HIV-seropositive individuals were newly diagnosed, of whom 557 qualified individuals were willing to participate in this study. Among them, 267 individuals started antiviral therapy within 1 year after the initial diagnosis, and 207 completed the study investigation, for a response rate of 77.5%.

### Data collection

Participants who started free antiretroviral therapy completed face-to-face interviews when they attended the CDC clinic or the Infection Disease Hospital. During medical visits, information was collected by trained investigators using a standard set of questionnaires. Clinical information, including baseline CD4+ cell counts and the antiretroviral therapeutic regimen, were collected from medical records. All interviews were conducted within 6 months after the antiretroviral therapeutic regimen was determined.

### Measurements

#### General information

Self-designed questions were used to collect demographic information, including sex, age, marital status, educational status, household registration, monthly income, living conditions and employment status. Clinical information (including baseline CD4 counts, route of infection, AIDS-related clinical symptoms, such as weight loss in the last 3 months, tuberculosis, or diarrhea) and antiviral therapy information (including the therapeutic regimen, side effects, and medication reminders) were collected from the medical records of the Chinese HIV/AIDS Comprehensive Response Information Management System.

#### Medication adherence

Adherence to medication was assessed with the Community Programs for Clinical Research on AIDS (CPCRA) Antiretroviral Medications Self-Report Questionnaire [28]. Participants were asked to recall the times and amounts of missed medications during the past week, and the ratio of medication adherence (dosage taken) was calculated. Ratios equal to or greater than 95% were defined as good dosage adherence, whereas ratios below 95% were defined as poor dosage adherence [6]. In addition, participants were asked to report the actual medication intake time and the prescribed intake time over the past week (dose timing). During the past week, a time difference within 1 h at every dose taken was defined as good time adherence (adherence to the medication intake interval), whereas a time difference greater than 1 h was defined as poor time adherence [9, 29]. In this study, good adherence was defined as a combination of good dosage (≥95%) and time adherence (within 1 h), all other situations was categorized as poor adherence.

#### Emotional problems

Depression and anxiety were measured with the Patient Health Questionnaire-9 (PHQ-9) [30] and the 7-item Generalized Anxiety Disorder (GAD-7) [31], respectively, using four-point scales with scores ranging from 0 (rarely) to 3 (most of the time). For the PHQ-9, scores of 5 to 9, 10 to 14, 15 to 27 indicated mild, moderate, and severe depressive symptoms, respectively. For the GAD-7, scores of 5 to 9, 10 to 14, and 15 to 21 indicated mild, moderate, and severe anxiety symptoms, respectively. A score ≥ 10 for the PHQ-9 and GAD-7 indicated the presence of positive depression and anxiety symptoms, with higher scores indicating more severe symptoms. Both scales have high reliability and validity [32, 33].

#### Knowledge of ART

Knowledge of ART was measured using a five-item questionnaire [19], including general knowledge of the side effects, the effects of ART, and the importance of adherence to ART. Knowledge was calculated by adding the number of correct answers to the questions; one point was given for each correct answer, and 0 points was given for each incorrect or uncertain answer. The total score ranged from 0 to 5. Higher scores indicated higher levels of knowledge of ART.

#### Satisfaction with medical services

Satisfaction with medical services was measured by a subjective question and was assessed on a 0–10 scale, where 0 was not satisfied at all, and 10 was completely satisfied. Participants were asked, “Overall, your satisfaction with the treatment services provided by the medical staff at the treatment site is…”.

### Data analysis

Data were processed using SPSS version 18.0 (SPSS Inc., Chicago, IL, USA). Sample characteristics were described with the frequency, mean, and standard deviation (SD). Univariate analyses were made by chi-square/ t-test but no univariate screening. Multivariable regression model were conducted by multivariate logistic regression analysis with backward stepwise. Medication adherence which measured within 3–6 months of receiving antiviral treatment was dived into two levels (good adherence = 0, poor adherence = 1) as the dependent variable. Based on professional judgement, 19 variables (which we have evaluated in this study) potentially associated with adherence were chosen as the independent variables, which included socio-demographic characteristics (sex, age, household registration, education level, marital status, monthly income, living status, employment status), HIV-related information (infection route, disclosure of HIV status, CD4 count, AIDS-related symptoms), psychosocial factors (depression symptoms, anxiety symptoms), antiviral treatment information (therapeutic regimen, side effects and medication reminders), knowledge of ART and satisfaction with medical services. Multicollinearities were tested before modeling, with VIF ranging from 1.09 to 2.06. The final logistic regression model was statistically significant (*P* = 0.000, χ^2^ = 18.787), the goodness-of-fit test also showed good model fit (*P* = 0.409, χ^2^ = 8.255), and the model’s percentage accuracy in classification was 84.8%. The significant level of selected variables was 0.05 (α = 0.05), and the significant level of elimination variables was 0.10 (α = 0.10). *P*-values less than 0.05 were considered significant.

## Results

### Participant characteristics

Of the 207 participants who completed the follow-up survey, 88.9% were male, 49.8% were unmarried, and the mean age was 35 years (SD = 12), with a range from 18 to 76 years. Half of the participants (49.8%) had household registrations in urban areas, 38.6% had received a college degree or above, 63.3% were employed, and 53.2% had a monthly income of less than 3500 yuan. A total of 24.6% (*n* = 51) of the participants reported living alone. The vast majority of the participants (98.3%, 174/177) used medication reminders. The most common reminders were an alarm clock (52.5%) and cell phones (41.5%), followed by reminders from family (6.3%). The majority of the participants (97.1%) were infected through sexual transmission, of which heterosexual infections accounted for 49.8% and homosexual infections accounted for 47.3% of the cases. The mean of baseline CD4^+^ cell count was 298/mm^3^ (SD = 159). 69.6% (144/207) of the patients reported side effects after taking the prescribed medication, and the most common side effects were dizziness/headache (39.7%), followed by appetite change (20.1%) and hypomnesis (18.6%). All participants were prescribed a first-line regimen, which was mainly composed of two nucleoside reverse transcriptase inhibitors (NRTIs) and one non-nucleoside reverse transcriptase inhibitor (NNRTI) or two NRTIs and one enhanced protease inhibitor (PI refers to Lopinavir/Ritonavir). Most patients (73.4%) were prescribed Tiroflove, lamivudine, and efavirenz, of which 39.7% (60/150) reported dizziness/headache, 18.7% (28/151) reported hypomnesis and 17.2% (26/151) reported appetite change. A total of 38.6% of the patients had AIDS-related clinical symptoms, of which “weight loss over 10% in the last three months” (15.5%) and “diarrhea for more than one month” (6.8%) were most commonly reported (Table 1).

**Table 1:** Factors associated with medication adherence among newly treated HIV-infected patients

Characteristics	Total N(%)	Good adherence n(%)	t/χ2	OR(95%CI)
Socio-demographic characteristics				
Sex			0.702	–
Male	184 (88.9)	156 (84.8)		
Female	23 (11.1)	21 (91.3)		
Age (years)			0.347	–
18–28	79 (38.2)	69 (87.3)		
≥ 28	128 (61.8)	108 (84.4)		
Household registration			0.179	–
Rural	104 (50.2)	90 (86.5)		
Urban	103 (49.8)	87 (84.5)		
Education level			0.418	–
Senior or lower	127 (61.4)	107 (84.5)		
College or higher	80 (38.6)	70 (87.5)		
Marital status			0.004	–
Single	103 (49.8)	88 (85.4)		
Divorced/Widowed	34 (16.4)	29 (85.3)		
Married	70 (33.8)	60 (85.7)		
Monthly income(yuan)*			0.051	–
≤ 3500	107 (53.2)	91 (85.0)		
> 3500	94 (46.8)	81 (86.2)		
Living alone			0.032	–
Yes	51 (24.6)	44 (86.3)		
No	156 (75.4)	133 (85.3)		
Employment status			1.525	–
Unemployed	76 (36.7)	68 (89.5)		
Employed	131 (63.3)	109 (83.2)		
HIV-related information				
Infection route			2.876	–
Blood/Uncertain	6 (2.9)	6 (100.0)		
Homosexual transmission	98 (47.3)	80 (81.6)		
Heterosexual transmission	103 (49.8)	91 (88.3)		
Disclosure of HIV status			2.381	
No	71 (34.3)	57 (80.3)		2.62 (1.06–6.50)*
Yes	136 (65.7)	120 (88.2)		Ref
CD4 count(mm^3^)*				
≤ 350	147 (71.4)	124 (84.4)	0.484	–
> 350	59 (28.6)	52 (88.1)		
AIDS-related symptoms			9.017**	–
No	127 (61.4)	116 (91.3)		
Yes	80 (38.6)	61 (76.2)		
Psychosocial factors				
Depressive symptoms			20.248**	
Yes	139 (67.1)	113 (81.2)		5.95 (2.34–15.11)*
No	68 (32.9)	64 (94.1)		Ref
Anxiety symptoms			12.279**	–
Yes	133 (64.3)	109 (81.9)		
No	74 (35.7)	68 (91.9)		
Antiviral treatment information				
Therapeutic regimen^**#**^			4.2	–
AZT + 3TC + EFV	30 (14.5)	26 (86.7)		
Other^a^	25 (12.1)	18 (72.0)		
TDF + 3TC + EFV	152 (73.4)	133 (87.5)		
Side effects			0.235	–
Yes	144 (69.6)	122 (84.7)		
No	63 (30.4)	55 (87.3)		
Medication reminders*			0.772	–
0	3 (1.7)	2 (66.7)		
1	151 (85.3)	131 (86.8)		
≥ 2	23 (13.0)	17 (73.9)		
Knowledge of ART (M ± SD)		1.68 ± 0.90	1.107	–
Satisfaction with medical services (M ± SD)		9.38 ± 1.11	2.976	–

*P < 0.05; **P < 0.01

*OR* Odds ratio; —:not included in the final model

#*TDF* Tiroflove, *3TC* Lamivudine, *EFV* Efavirenz, *AZT* Zidovudine, *NVP* Nevirapine, *LPV/r* Lopinavir/Ritonavir

^a^AZT + 3TC + NVP, AZT + 3TC + LPV/r, 3TC + NVP + TDF;

*Ref* Reference group; * data missing

### Medication adherence

Among the 207 participants, 87.0% (180/207) reported good dosage adherence, 87.4% (181/207) reported good time adherence, of which 94.5% (171/181) indicated that the interval between medication was 5 min in the past week. In total, 85.5% of the participants (177/207) were categorized with good adherence, and 14.5% (30/207) with poor adherence.

### Emotional problems and disclosure of HIV status

The mean PHQ-9 and GAD-7 scores were 8.77 (SD = 6.74) and 7.26 (SD = 5.78), respectively. Among the 207 participants, 41.1% (85/207) had positive depressive symptoms; the rates of mild, moderate and severe depressive symptoms were 26.1%,16.9%, and 24.2%, respectively. Additionally, 29.5% of the participants (61/207) had positive anxiety symptoms; the rates of mild, moderate and severe anxiety symptoms were 34.8%,15.5%, and 14.0%, respectively (Table 1). There were 34.3% (71/207) had not disclosed their HIV status to others (including family, partners, relatives, and friends), of whom 74.1% (53/71) reported had positive depressive symptoms, which was higher than those had disclosed (63.2%) (*P* = 0.046, χ^2^ = 2.754).

### Knowledge of ART and satisfaction with medical services

Of the 207 participants, all but one completed these questions, and 7.7% (16/207) answered all of the questions incorrectly. The mean score for the knowledge of ART was 1.65 (SD = 0.78). A total of 67.1% of the participants were completely satisfied with the medical services they received. The mean score for satisfaction with medical services was 9.34 (SD = 1.13) (Table 1).

### Factors related to medication adherence

The univariate analysis showed that participants with AIDS-related symptoms, having positive anxiety and depressive symptoms were more inclined to show poor adherence. Please see Table 1. The multivariate logistic regression analyses showed that participants with positive depression (OR = 5.95, 95% CI: 2.34–15.11) and without disclosure of their HIV status to others (OR = 2.62, 95% CI: 1.06–6.50) were more susceptible to poor adherence (Table 1).

## Discussion

The rate of dosage adherence among newly received antiretroviral therapy (within 3–6 months) was 87.0% in this study. This rate was higher than the result obtained by Wang [34], who reported 78.9% dosage adherence rate among patients with HIV infections with an average treatment duration of 17.7 months but was lower than the results of Xiao [35], who reported a 96.6% dosage adherence rate. This difference might due to the different characteristics and treatment cascades of the samples, since we compared the rate using the same operational definitions of adherence behavior within the studies. The rate of taking the medicine within the prescribed time was 87.4%, which was higher than the rate indicated by Gill [29], who reported 47.8% dosing time adherence rate in the past months. This might due to the inconsistent time of medication length, the medication adherence would decrease with the medication time increasing [7]. It is suggested that more attention should be paid to the adherence of newly treated people living with HIV.

The univariate analysis revealed that participants with positive anxiety symptoms, positive depression symptoms and AIDS-related symptoms had a higher tendency for poor adherence, which were consistent with previous studies [19, 23, 26]. Globally, numerous studies have demonstrated that people living with HIV are vulnerable to psychological problems [24, 36–39]. This group faces a high risk of mental health problems, but the psychosocial needs of HIV-positive individuals remain largely unaddressed in China [40]. The diagnosis of HIV status is a negative life event that results in negative psychological reactions, such as depression, anxiety and suicidal ideation [38, 41, 42]. Newly treated people living with HIV, especially those with AIDS-related symptoms, not only have to accept the fact of chronic condition, but also have to comply with complex medication regimens, which usually leads to increased symptoms of anxiety and depression. Additionally, participants with positive anxiety and depression may be more likely to have physiological symptoms that are attributed to the side effects of the antiviral regime [36, 43]. In the final model, this study did not demonstrate an association between anxiety and medication adherence (although 64.3% of the participants had anxiety symptoms, and 14.0% had severe anxiety symptoms). This result might due to the relatively short follow-up duration of the antiviral therapy for the study sample. Anxiety might worsen with a prolonged treatment time and decreased physical functioning of the patients, eventually resulting in adverse influences on medication adherence behavior [44]. Future interventions to facilitate patients’ adherence to antiretroviral therapy should pay close attention to their emotional health and changes in their emotional health over time. Furthermore, effective psychological coping strategies must be provided for this group.

Participants who refused to disclose their HIV status had poor adherence, which was consistent with previous studies [45].The reason may be that non-disclosure impeded them from obtaining social support. Patients with lower social support were more inclined to show poor adherence [46]. In this study, the positive rates of depression among those who refused to disclose their HIV status were higher than those of the disclosers, this also might cause the negative association between non-disclosure and adherence. In addition, participants who did not disclose their HIV status to others put more pressure on themselves in terms of taking regular medication [47], which might have led to missing dose intentionally especially for those living with others. We can reasonably assume that self-discrimination and fear of the deterioration of the relationship may have interfered with their disclosure to others, which in turn negatively affected their adherence. Thus, adherence-enhancing interventions for people living with HIV should consider incorporating psychosocial coping strategies, including providing assistance for disclosure to supportive others, improving their utilization of social support from their surroundings, and developing practical tips on how to keep their privacy while taking medication in the right amount at the right time in different situations may also be an important approach.

### Limitations of the study

This study had several limitations. First, although the sample was obtained through longitudinal observation and the assessment of adherence was cross-sectional, this evidence was not sufficient to infer causality. Second, adherence data were gained by self-report, which might result in reporting bias as well as the respondents’ recollection bias and the tendency to overestimate medication adherence. In addition, we used the Community Programs for Clinical Research on AIDS Antiretroviral Medications and Self-Report Questionnaire (CPCRA) to assess patients medication adherence for the past 7 days, short-term medication adherence may be influenced by ***“***white coat***”*** effect. Patients showed better adherence before interview, it may also lead to overestimation of medication adherence [48]. Subsequent studies should strive to innovate in these areas by adopting a longitudinal study design and developing more objective adherence measurement methods.

## Conclusions

One-sixth of the participants reported suboptimal medication adherence within the first 6 months. Factors associated with poor adherence included non-disclosure of their HIV status, had positive depression. Tailored interventions, such as assistance with an appropriate disclosure process and effective medication reminder strategies involving family or other cohabitants in the psychosocial support program, should be implemented to improve medication adherence during established ART.

## Abbreviations

3TC: Lamivudine
ART: Antiretroviral therapy
AZT: Zidovudine
CD4 +: Cluster of differentiation 4
EFV: Efavirenz
HIV/AIDS: Human immunodeficiency virus/Acquired immunodeficiency syndrome
LPV/r: Lopinavir/Ritonavir
NNRTI: Non-nucleoside reverse transcriptase inhibitor
NRTIs: Nucleoside reverse transcriptase inhibitors
NVP: Nevirapine
PI: Protease inhibitor
SPSS: Statistical product and service solutions
TDF: Tiroflove
